# The HIV pre-exposure prophylaxis continuum of care among women who inject drugs: A systematic review

**DOI:** 10.3389/fpsyt.2022.951682

**Published:** 2022-08-26

**Authors:** Danielle Guy, Jason Doran, Trenton M. White, Lena van Selm, Teymur Noori, Jeffrey V. Lazarus

**Affiliations:** ^1^Barcelona Institute for Global Health (ISGlobal), Hospital Clínic, University of Barcelona, Barcelona, Spain; ^2^National Infection Service, UK Health Security Agency, London, United Kingdom; ^3^London School of Hygiene and Tropical Medicine, University of London, London, United Kingdom; ^4^European Centre for Disease Prevention and Control, Stockholm, Sweden; ^5^Faculty of Medicine and Health Sciences, University of Barcelona, Barcelona, Spain

**Keywords:** pre-exposure prophylaxis (PrEP), PrEP care continuum, women who inject drugs, human immunodeficiency virus, people who inject drugs

## Abstract

**Introduction:**

People who inject drugs have a substantial risk for HIV infection, especially women who inject drugs (WWID). HIV pre-exposure prophylaxis (PrEP), a highly-effective HIV prevention drug, is uncommonly studied among WWID, and we aimed to synthesize existing knowledge across the full PrEP continuum of care in this population.

**Methods:**

We systematically searched for peer-reviewed literature in three electronic databases, conference abstracts from three major HIV conferences, and gray literature from relevant sources.

Eligibility criteria included quantitative, qualitative or mixed-methods studies with primary data collection reporting a PrEP-related finding among WWID, and published in English or Spanish between 2012 and 2021. The initial search identified 2,809 citations, and 32 were included. Data on study characteristics and PrEP continuum of care were extracted, then data were analyzed in a narrative review.

**Results:**

Our search identified 2,809 studies; 32 met eligibility requirements. Overall, awareness, knowledge, and use of PrEP was low among WWID, although acceptability was high. Homelessness, sexual violence, unpredictability of drug use, and access to the healthcare system challenged PrEP usage and adherence. WWID were willing to share information on PrEP with other WWID, especially those at high-risk of HIV, such as sex workers.

**Conclusions:**

To improve PrEP usage and engagement in care among WWID, PrEP services could be integrated within gender-responsive harm reduction and drug treatment services. Peer-based interventions can be used to improve awareness and knowledge of PrEP within this population. Further studies are needed on transgender WWID as well as PrEP retention and adherence among all WWID.

## Introduction

Injecting drug use is a major driver of HIV infection globally, with up to ten percent of HIV infections attributable to injecting drug use (1). People who inject drugs (PWID) are 22 times more likely to acquire HIV compared to those who do not, and one in every eight individuals who injects drugs is living with HIV (1, 2). Women who inject drugs (WWID) are particularly at increased risk of HIV infection compared to men, primarily as a consequence of high-risk injection and sexual practices, such as sharing needles and engaging in condomless sex (3). This is due to a variety of structural factors including the criminalization of drug use, which disproportionately affects women who use drugs (4), gendered injecting practices, such as women being forced to share needles (5), and gender-based violence, which is associated with high-risk sexual behaviors and avoidance of health services among WWID (3). Additionally many WWID participate in transactional sex or sex work, which is associated with higher rates of HIV due to gendered power dynamics, which increase women's exposure to sexual violence and limit their abilities to negotiate safe sex (4, 6).

HIV risk among WWID is compounded by the intersection of stigma related to both substance abuse and gender particularly due to gendered expectations of morality and motherhood (7). This stigma impacts WWID within and outside of injecting communities (8). Outside of injecting communities, this stigma can diminish trust in the health system and health providers, which may decrease health-seeking behaviors and access to HIV-related and other health services, including harm reduction services (5, 8, 9). Moreover, harm reduction services for people who inject drugs (PWID), are often male-oriented, meaning they serve primarily male clientele and lack the staff or facilities to address the distinct needs of women (5). As a consequence, WWID often do not have their unique needs met in these settings and may be forced to engage in unsafe injecting.

A possible solution to decrease both injection and sexual-related HIV risk among WWID is pre-exposure prophylaxis (PrEP) (10). PrEP is highly effective in preventing HIV (11–13), and its use has been expanding rapidly since the World Health Organization (WHO) recommended it for high-risk populations in 2015 (14, 15). By 2019, 180 countries had adopted these recommendations, but with only an estimated 626,000 PrEP users in only 77 countries, primarily in North and South America and sub-Saharan Africa (16).

Even in high-income countries, WWID are not identified as a priority group for PrEP interventions. Effective PrEP interventions should consider all high-risk populations and include the full PrEP continuum of care, including PrEP initiation, adherence, and retention or disengagement in care (17). While there is a growing number of studies on WWID and PrEP, there is no synthesis of the current evidence base. One study previously examined the PrEP care cascade among PWID, but it only focused on the US and did not examine gender differences (18). A global review of the PrEP care cascade focused more broadly on women who use drugs and female sex workers (19). However, it did not consider transgender women, who are at higher risk of HIV (2), it did not consider the full PrEP cascade, and it only included peer-reviewed literature. As such, the aim of this study is to examine the entire PrEP continuum of care among women (cis and trans) who inject drugs globally.

## Methods

**Figure 1:**
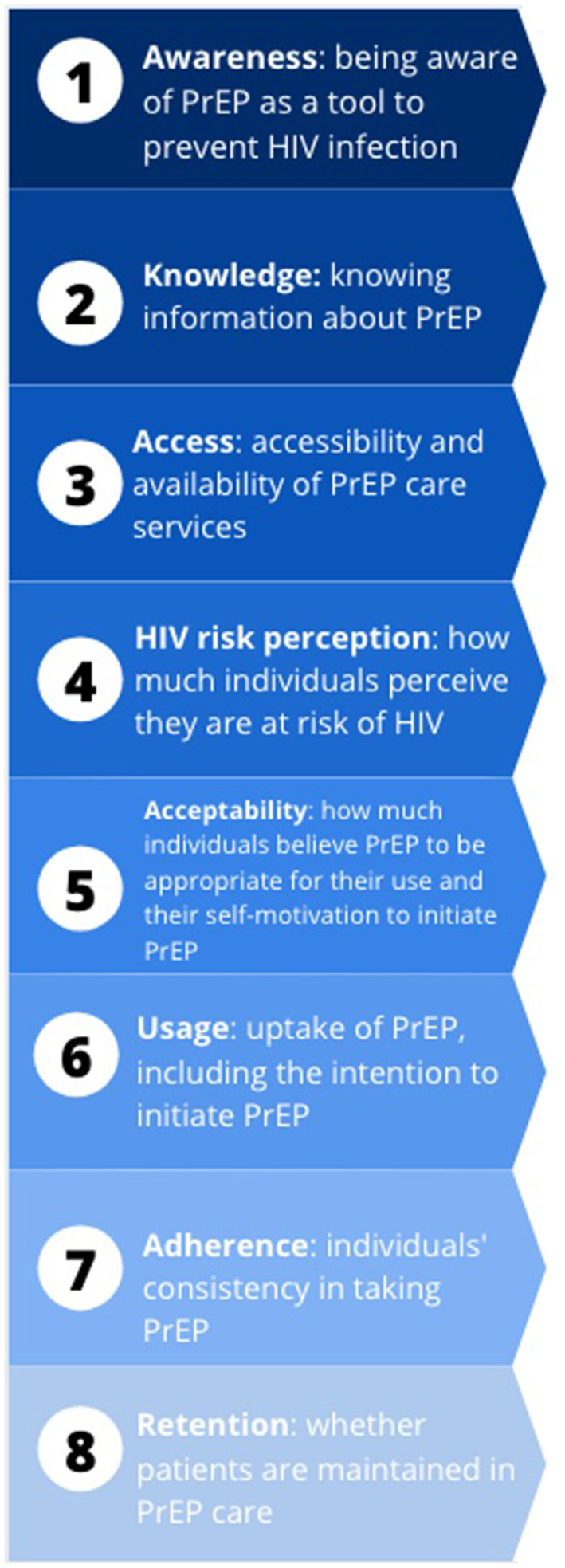
PrEP continuum of care variables and definitions Nunn et al. (17)

We reviewed studies that considered any part of the PrEP care cascade among women (cis and trans) who inject drugs globally. For each study, we analyzed at least one of the following variables, based on the framework by Nunn et al. (17): PrEP awareness, PrEP knowledge, access to PrEP care, HIV risk perception, PrEP acceptability, PrEP usage, PrEP adherence, or retention in PrEP care (see Figure 1) or any other relevant PrEP variables.

### Included studies and search strategy

Any quantitative, qualitative, or mixed-methods study with primary data collection was eligible for inclusion. We did not include commentaries, editorials, or reviews. However, the bibliographies of relevant reviews were searched for relevant articles for inclusion. Publications must have been published from 2012 onwards, when PrEP was first approved by the US Federal Drug Administration to prevent HIV in at-risk populations. We only included publications which focused specifically on women or studies that present gender differences of relevant results. All publications must have been reported in either English or Spanish.

A comprehensive literature search was completed in PubMed/Medline, CINAHL, and PsycINFO. The search string included subject headings and keywords related to HIV and PrEP, the PrEP care continuum, injecting drug use, and gender/sex (see Table 1). We reviewed the references of included papers to check for other relevant studies. We also searched for abstracts in three major, relevant conference proceedings: the International AIDS Conference, HIV Research for Prevention conference, and the International AIDS Society Conference on HIV Science. Additionally, we searched clinicaltrials.gov, the WHO's International Clinical Trials Registry Platform, and for additional gray literature from Harm Reduction International, the Women in Harm Reduction International Network, the International Network of People Who Use Drugs, the International Drug Policy Consortium, Correlation European Harm Reduction Network, and the New York Academy of Medicine's Gray Literature Database.

**Table 1:** Search strategy (PubMed 17 June 2021).

**Search**	**Query**	**Results (since 2012)**
#1	[“HIV” AND (“care” OR “risk” OR “prevention” OR “service”)] OR “PrEP” OR “Pre-Exposure prophylaxis”[Mesh] OR “treatment as prevention” OR “TasP” OR “Pre Exposure Prophylaxis” OR “pre-exposure chemoprophylaxis*” OR “pre-exposure antiretroviral prophylaxis” OR “Antiretroviral chemoprophylaxis” OR “Truvada”	88,161
#2	“Injecting drug use*” OR “Intravenous drug use*” OR “People who inject drugs” OR “Women who inject drugs” OR “Women who use drugs”OR “PWID” OR “WWID” OR “Addict*” OR “IDU” OR “People who use drugs” OR “PWUD” OR “Substance Abuse, Intravenous“[Mesh]	73,573
#3	“Patient compliance”[Mesh] OR “Medication Adherence”[Mesh] OR “Attitude to Health”[Mesh] OR “Compliance” OR “Access” OR “Adherence” or “Perception” OR “Non-compliance” or “Non-adherence” or “Attitude” OR “Acceptability” OR “Feasibility” OR “Retention” OR “Engagement” OR “Disengagement” OR “Usage” OR “Uptake” OR “Willingness” OR “Initiation” OR “Knowledge” OR “Availability”	1,713,855
#4	“Women”[Mesh] OR “Female”[Mesh] OR “Women's Health”[Mesh] OR “Women's Health Services”[Mesh] OR “Sex”[Mesh] OR “Sex Characteristics”[Mesh] OR “Sex Distribution”[Mesh] OR “Gender Identity”[Mesh] OR “Women” OR “Gender” OR “Sex” OR “Trans women” OR “Female”	3,460,850
#5	#1 AND #2 AND #3 AND #4	1,573

### Data extraction and synthesis

All records were imported into Mendeley and duplicated records were removed. Two reviewers (DG and TMW) screened titles and abstracts of records identified through the search strategy, and disagreements were resolved between the two. Full texts of records were assessed for inclusion by two reviewers (DG and JD); disagreements were resolved with another reviewer (TMW).

Data were extracted by two reviewers (DG and LvS) into a pre-specified data extraction template in Microsoft Excel. Information included authorship, title, study aims, design, setting, population, sample size, PrEP care continuum findings, and other relevant findings. Data were then validated by another reviewer (TMW) and differences were reconciled among two reviewers (DG and TMW). Narrative synthesis, organized according to each step of the PrEP care cascade, was performed to describe the characteristics and findings of all included studies.

### Risk of bias assessment

Two reviewers (DG and LvS) assessed risk of bias for each study individually and when scores differed, discrepancies were resolved through discussion between the two reviewers. Risk of bias was assessed using the 2018 Mixed Methods Appraisal Tool (MMAT) (20), which allows for evaluation of qualitative, quantitative, and mixed method studies. Each study received a quality score ranging from 0 (meeting no criteria) to 5 (meeting all criteria) based on the MMAT criteria.

## Results

**Figure 2:**
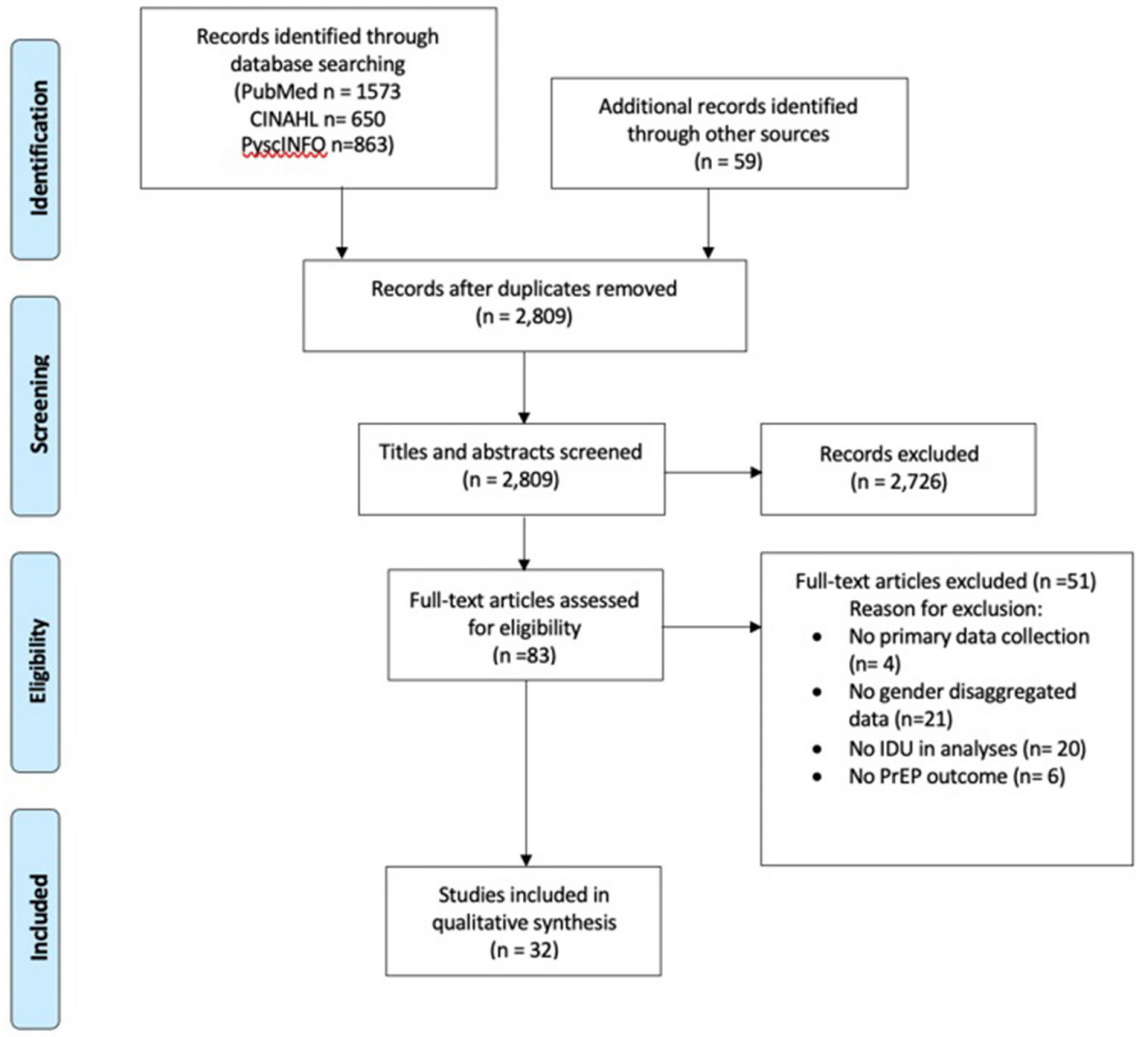
PRISMA flow diagram.

The initial search yielded 3,145 records, and 2,809 remained after removing duplicates. After screening titles and abstracts, 83 articles remained to be assessed for eligibility at the full-text level. Fifty-one articles were excluded in total. Articles were excluded because they did not provide gender disaggregated results (*n* = 21), did not include injecting drug use in their analyses (*n* = 20), did not show PrEP outcomes (*n* = 6), or did not use primary data collection (*n* = 4; Figure 2).

### Characteristics of included studies

Thirty-two studies were included in this review (see Table 2). Studies primarily took place in the United States (21, 22, 26–31, 35, 36, 39–47, 49–51) but also in Thailand (37, 38), Canada (25), India (24), Kenya (23), and Malaysia (32). One study included participants globally (34) and another included those in Europe and Asia only (33). Study populations were primarily adult (18+) PWID (24, 25, 33–39, 43, 44, 46, 47, 52) or WWID only (22, 23, 26–31, 49, 50). Other populations included female sex workers (41), transgender women (32), prison inmates (21), individuals at high-risk of HIV (51), women with substance abuse disorders (42), and opiate users (40, 48). Sample sizes ranged from 9 (23) to 10,538 (24) participants. Across the studies, where sample sizes of WWID were provided, 3,216 WWID were included. The average quality score for the studies was 4.2, and studies were not excluded based on quality rating (see Table 3).

**Table 2:** Characteristics of included studies.

**References**	**Study design**	**Setting**	**Population (sample size)**	**PrEP variables**	**Findings**
Alarid and Hahl (21)	Cross-sectional survey	United States	Prison inmates (*N* = 595; *n* = 260 women)	HIV risk perception	IDU was positively associated with the perceived risk of HIV seroconversion.
Bass et al. (22)	Focus groups	United States	WWID using a large urban syringe exchange (*N* = 24)	Prep awareness PrEP acceptability PrEP usage	Sixty-six percentage of WWID were aware of PrEP; 41.6% of WWID initiated PrEP; Most (unclear how many) were interested in PrEP, but seeing a doctor was a significant barrier. Other barriers were homelessness and potential theft of medication. Facilitators of PrEP use could include providing it at the syringe exchange, providing it on a daily basis and in pill packs.
Bazzi et al. (23)	Interviews	Kenya	HIV-uninfected WWID (*N* = 9)	PrEP awareness PrEP acceptability	Only one woman had heard of oral PrEP. Generally, acceptability was high, but women were concerned about unknown side effects and efficacy. One woman was concerned about not being able to tolerate PrEP during drug withdrawal. Another woman had concerns about the increase of condomless sex with the use of PrEP and STIs. Drug use was not a significant deterrent to adopting or adhering to PrEP.
Belludi et al. (24)	Questionnaire	India	PWID (*n* = 10,538; *n* = 313 WWID) and MSM (*n* = 8,621)	PrEP awareness HIV risk perception PrEP acceptability	Gender was not associated with willingness to use PrEP in adjusted and unadjusted analyses. Sixty-two percentage of WWID were willing to use PrEP, 29% did not endorse self-perceived HIV risk as a reason for unwillingness; 9% endorsed a lack of self-perceived HIV risk for unwillingness.
Corcorran (52)	Cross-sectional survey	United States	PWID at syringe service providers (*N* = 348; *n* = 130 WWID)	PrEP awareness PrEP acceptability	Gender was not associated with willingness to use PrEP. Fifty-six percentage of women were aware of PrEP. Correlates of interest included being high-risk for HIV (i.e., meth/heroin use, exchange sex, and experiencing homelessness, and sharing injection equipment), and being PrEP aware.
Escudero et al. (25)	Questionnaire	Canada	HIV-negative PWID (*N* = 543; *n* = 166 WWID)	PrEP acceptability	Forty-four percentage of WWID were willing to use PrEP. More WWID were willing to use PrEP compared to their male counterparts [OR 1.52 (1.05–2.22) *p* = 0.028]. Side effects of PrEP was a main barrier to PrEP acceptability. Willingness to use PrEP was also positively correlated with younger age [adjusted odds ratio (AOR) = 1.30 per 10 years younger; 95% CI: 1.05–1.59], no regular employment (1.67; 1.05–2.65), requiring help injecting (2.14; 1.11–4.11), sex work (2.29; 1.01–5.20), and multiple recent sexual partners (2.00; 1.07–3.74).
Felsher et al. (26)	Cross-sectional survey	United States	HIV-negative, cisgender WWID at a syringe service provider (*N* = 89)	PrEP usage	77.5% of women initiated PrEP. PrEP initiation was significantly associated with reporting sexual assault (*p* = 0.003), higher income (*p* = 0.06), frequency of SSP attendance (*p* = 0.001), and inconsistent condom use (*p* = 0.03).
Felsher et al. (27)	Interviews	United States	HIV-negative WWID participating in a PrEP demonstration project (*N* = 20)	PrEP communication	PrEP conversations occurred within 30/57 relationships. Motivations for communication were to benefit others (enabled by HIV risk, gender similarity, perception of peer at risk of HIV, little negative outcomes expected from discussion), benefit themselves (to increase emotional connectedness and potential support from a peer), or perceived obligation (negative outcome perceived from not disclosing PrEP use when in a shared living space).
Felsher et al. (28)	Interviews	United States	WWID (*N* = 25)	HIV risk perception PrEP acceptability	Most WWID were concerned about HIV risks related to sexual assault and environmental forces beyond their control (e.g., accidental needle sticks). WWID who had regular engagement in harm reduction behaviors (e.g., avoiding syringe sharing) perceived themselves to be at low risk of HIV. WWID unanimously perceived PrEP to be a beneficial HIV prevention tool. Potential adverse reactions with comorbid conditions, PrEP- and HIV-related stigma, location of care, and the psychological costs of initiating new relationships with PrEP care providers influenced PrEP acceptability.
Felsher et al. (29)	Social network survey	United States	WWID (*N* = 40)	HIV risk perception PrEP usage PrEP communication	47.5% of WWID perceived themselves as at high risk of HIV. Nearly all (97.5%) accepted a PrEP prescription. 83.2% of WWID were willing to share PrEP information. Participants were more likely to share PrEP information with individuals who were homeless (UOR 3.3; 95% CI 1.5–7.6), an injecting drug user (UOR 2.3; 95% CI 1.1–4.7), engaged in transactional sex (UOR 4.5; 95% CI 1.6–12.5) or had a perceived high-risk of HIV (UOR 1.1; 95% CI 1.1–1.2).
Felsher et al. (30)	Interviews	United States	WWID (*N* = 23)	PrEP adherence	Only 5.6% of WWID were adherent to PrEP. PrEP ranked relatively low compared to other basic needs. Women's perceived need for PrEP fluctuated with their drug use and HIV risk perception. Women who did not have stable housing often described how the lack of safe pill storage leads to pills being lost or stolen.
Footer et al. (31)	Focus groups	United States	WWID (*N* = 16)	PrEP awareness PrEP knowledge	31% of WWID were aware of PrEP. Knowledge was “low” but not quantified. All WWID were interested in PrEP as an additional form of HIV protection. Women had concerns about convenience and ease of use, preferring less frequent delivery methods. Potential interactions with other medication regimens and access to medical providers were noted as concerns about PrEP use.
Galka et al. (32)	Cross-sectional survey	Malaysia	Transgender women (*N* = 361 total; *n* = 10 WWID)	PrEP awareness PrEP acceptability	In the bivariate analysis, IDU was significantly associated with lower willingness to use PrEP {OR −1.17 [95% CI (−1.85 to −0.48), *p* = 0.001]}. But in the multivariable analysis, the difference was not significant (*p* = 0.041).
International Network of People who Use Drugs (33)	Interviews and face-to-face consultations	Europe and Asia	PWID (*N* = 75 total; *n* = 23 WWID)	PrEP access, PrEP acceptability	Criminalization of drug use and stigma toward people who inject drugs negatively affect the accessibility of PrEP. Scale up of harm-reduction services, especially community-based services would be necessary for PrEP to be accessible to participants. Participants recognized the potential benefit of PrEP and emphasized its use in a larger package of comprehensive services. But participants generally preferred access to safe injection equipment than using a daily pill to prevent HIV infection.
International Network of People who Use Drugs (34)	Semi-structured interviews and focus groups discussions	Global	PWID (*N* = 54 total; *n* = 17 WWID)	PrEP knowledge, access to PrEP, PrEP, PrEP acceptability	Few participants expressed that they did not feel sufficiently informed on PrEP. Several participants noted issues with PrEP availability and highlighted the ethical issue of making individuals aware of PrEP without allowing them access to it, particularly for modes of PrEP relevant to women (e.g., vaginal rings). Participants were generally willing to use PrEP but underscored the necessity for it to be a part of a comprehensive package of harm reduction services. Several participants highlighted the issue of lack of basic harm reduction services, such as lack of safe injection equipment, which was more pressing than PrEP.
Jo et al. (35)	Cross-sectional survey	United States	PWID at a syringe service provider (*N* = 157; *n* = 36 WWID)	PrEP acceptability	There was no statistically significant difference in the odds of expressing interest in PrEP by gender. In the adjusted model, people with opioid-only use were significantly less likely to report interest in being linked to PrEP.
Kuo et al. (36)	Interview and questionnaire	United States	PWID (*N* = 304; *n* = 98 WWID)	PrEP acceptability	38.7% of WWID were very likely to use PrEP and 27.5% were somewhat/not likely to use PrEP. Gender was not associated with willingness to use PrEP.
Martin et al. (37)	Randomized, double-blind, placebo- controlled, endpoint-driven study	Thailand	PWID (*N* = 2,413; *n* = 489 WWID)	PrEP adherence	47.7% of women had poor (<95% adherence). In the multivariable analysis, men were more likely to report poor adherence compared to women (*p* = 0.006).
Martin et al. (38)	Observational, cohort study	Thailand	Current or previous PWID (*N* = 1315; *n* = 274 WWID)	PrEP usage PrEP adherence Retention in PrEP care	Fifty-eight percentage of women chose to take PrEP. In the bivariate analysis, there was no significant difference in uptake by gender [OR 1.2 (95% CI 0.9–1.5)]. Sixty-nine percentage of WWID returned for at least one clinic visit, and gender was not significantly associated with attendance.
McFarland et al. (39)	Cross-sectional survey, data from National HIV Behavioral Surveillance (NHBS)	United States	PWID (*N* = 397 total, number of WWID not specified)	PrEP awareness PrEP knowledge PrEP usage PrEP communication	63.4% of WWID were aware of PrEP and women were more likely than men to be aware. 38.9% of WWID knew PrEP can prevent HIV transmission from sharing injection equipment, and there were no significant differences by gender. Only 13.6% of WWID discussed taking PrEP with their healthcare provider in last year. Three percentage of WWID used PrEP in last year. After excluding MSM, women were more likely to have used PrEP than men (3.7% of women vs. 0% of non-MSM men, *p* = 0.007).
Metz et al. (40)	Questionnaire	United States	Individuals with opioid use disorder (*N* = 138 total; *n* = 24 females)	PrEP awareness PrEP acceptability	Thirty percentage of the sample had heard of PrEP, with no significant differences between genders. PrEP acceptance was 59%, with no significant differences between genders. There were no gender differences in HIV risk behaviors, transmission and prevention knowledge or preferences.
Peitzmeier et al. (41)	Cross-sectional survey	United States	Female sex workers at a mobile health service (*N* = 60; *n* = 54 WWID)	PrEP awareness PrEP acceptability	Thirty-three percentage of WWID were aware of PrEP and 63% accepted PrEP. IDU was not significantly associated with increased interest in PrEP. Women that experienced physical or sexual violence from clients and women under 35 had higher PrEP acceptance.
Qin et al. (42)	Semi-structured interviews	United States	Women with substance use disorders (*N* = 20)	PrEP awareness	Thirty-five percentage of WWID were aware of PrEP. Motivations to engage in PrEP care were problematized by women's basic needs, lack of perceived risk of HIV, and anticipated stigma.
Roth et al. (43)	Cross-sectional survey	United States	PWID attending a syringe exchange program (*N* = 138 total; *n* = 65 WWID)	PrEP acceptability Access to PrEP	PrEP acceptance was higher in women compared to men (88.9 vs. 71.0%; *p* < 0.02). Few participants had accessed health services for HIV risk assessment that could lead to discussion about PrEP [at primary care physicians (43.8%), STI clinics (9.4%), or annual women's wellness examinations (15.4%)]. Most participants (86%) reported that they would prefer to access future screening at the syringe exchange program vs. traditional STI clinics.
Roth et al. (44)	Cross-sectional survey, data from National HIV Behavioral Surveillance (NHBS)	United States	PWID attending a syringe exchange program (*N* = 612; *n* = 155 WWID)	PrEP awareness	35.5% WWID were aware of PrEP. Factors associated with PrEP awareness were having at least some college education (aOR 2.13, 95% CI 1.03, 4.43), sharing paraphernalia (aOR 2.37, 95% CI 1.23, 4.56), obtaining syringes/needles primarily from a syringe exchange program (aOR 2.28, 95% CI 1.35, 3.87), STI testing (aOR 1.71, 95% CI 1.01, 2.89) and drug treatment (aOR 2.81, 95% CI 1.62, 4.87). Individuals that accessed prevention and health services had increased odds of being aware of PrEP.
Roth et al. (45)	Cross-sectional survey	United States	WWID attending a syringe service program (*N* = 136)	PrEP awareness PrEP usage PrEP adherence Retention in PrEP care	52.6% of participants were aware of PrEP before enrolling in the study. 63/95 initiated PrEP and uptake was associated with greater baseline frequency of SSP access (aOR = 1.85; 95% CI: 1.24–2.77), inconsistent condom use (aOR = 3.38; 95% CI: 1.07–10.7), and experiencing sexual assault (aOR = 5.89; 95% CI: 1.02, 33.9). 44.2% were retained in care at week 24, and retention was higher among women who reported more frequent baseline SSP access (aOR = 1.46; 95% CI: 1.04–2.24). Half the sample reported full adherence, but this was not confirmed by urinalysis.
Schneider et al. (46)	Survey	United States	PWID (*N* = 407; *n* = 159)	PrEP awareness PrEP acceptability PrEP usage	32.6% of WWID were aware of PrEP, 58.3% accepted PrEP, and 3.7% had used PrEP before. Acceptance per form of administration females: oral (62%), arm injection (60.1%), abdomen injection (21.6%), IV infusion (14.9%), under skin implant (26.4%) vaginal gels (26.6%) or vaginal rings (28.6%).
Sherman et al. (47)	Cross-sectional survey	United States	PWID (*N* = 265; *n* = 85 WWID)	PrEP acceptability	33.5% of WWID accepted PrEP, with no significant differences between men and women. PrEP interest was associated with being eligible for PrEP (aOR = 2.46; 95% CI: 1.34, 4.50) and the number of medical diagnoses (aOR = 1.16; 95% CI: 1.01, 1.33).
Stein et al. (48)	Cross-sectional survey	United States	Opiate users seeking opioid detoxification (*N* = 351; *n* = 105 WWID)	PrEP acceptability	50.5% of WWID were willing to use PrEP, and there were no differences in acceptability by gender. People who believed they were at risk for HIV had higher rates of acceptability.
Tran et al. (49)	Cross-sectional survey	United States	WWID (*N* = 95)	PrEP usage	Eighty-eight percentage of WWID intended to initiate PrEP. Overall, most WWID held positive attitudes about PrEP. Most (≥70%) had no concerns about PrEP's efficacy, and no/little concern about side-effects. There was no difference in PrEP intention between WWID who accepted PrEP and those who did not.
Walters et al. (50)	Cross-sectional survey, data from National HIV Behavioral Surveillance (NHBS)	United States	WWID (*N* = 118)	PrEP awareness	Thirty-one percentage of WWID were aware of PrEP. In multivariable logistic regression, increased PrEP awareness was associated with reported transactional sex (aOR 3.32, 95% CI 1.22–9.00) and having had a conversation about HIV prevention at a syringe exchange program (aOR 7.61, 95% CI 2.65–21.84).
Walters et al. (51)	Cross-sectional survey, data from National HIV Behavioral Surveillance (NHBS)	United States	Groups at high risk of HIV (*N* = 2,483; *n* = 196 WWID)	PrEP awareness Access to PrEP	Eighty percentage of WWID were aware of PrEP in Long Island and 12% in New York City. Among high-risk groups on New York City and Long Island, only 25 and 32% of WWID, respectively, had access to HIV prevention professionals.

**Table 3:** Risk of bias assessment using mixed methods appraisal tool.

**Qualitative studies**						
**References**	**Is the qualitative approach appropriate to answer the research question?**	**Are the qualitative data collection methods adequate to address the research question?**	**Are the findings adequately derived from the data?**	**Is the interpretation of results sufficiently substantiated by data?**	**Is there coherence between qualitative data sources, collection, analysis and interpretation?**	**Final score**
Bass et al. (22)	1	1	0	0	0	2
Bazzi et al. (23)	1	1	0	1	1	4
Felsher et al. (27)	1	1	1	1	1	5
Felsher et al. (28)	1	1	1	1	1	5
Felsher et al. (30)	1	1	1	1	1	5
Footer et al. (31)	1	1	1	1	1	5
International Network of People who Use Drugs (33)	1	0	1	1	1	4
International Network of People who Use Drugs (34)	1	1	1	1	1	5
Qin et al. (42)	1	1	1	1	1	5
**Quantitative randomized controlled trials**						
**References**	**Is randomization appropriately performed?**	**Are the groups comparable at baseline?**	**Are there complete outcome data?**	**Are outcome assessors blinded to the intervention provided?**	**Did the participants adhere to the assigned intervention?**	**Final score**
Martin et al. (37)	0	0	1	0	0	1
**Quantitative non-randomized controlled trials**						
**References**	**Is randomization appropriately performed?**	**Are the groups comparable at baseline?**	**Are there complete outcome data?**	**Are outcome assessors blinded to the intervention provided?**	**Did the participants adhere to the assigned intervention?**	**Final score**
Felsher et al. (26)	1	1	1	0	1	4
**Quantitative descriptive studies**						
**References**	**Is the sampling strategy relevant to address the research question?**	**Is the sample representative of the target population?**	**Are the measurements appropriate?**	**Is the risk of non-response bias low?**	**Is the statistical analysis appropriate to answer the research question?**	**Final score**
Alarid et al. (21)	1	0	1	1	1	4
Belludi et al. (24)	1	1	0	1	1	4
**References**	**Is randomization appropriately performed?**	**Are the groups comparable at baseline?**	**Are there complete outcome data?**	**Are outcome assessors blinded to the intervention provided?**	**Did the participants adhere to the assigned intervention?**	**Final score**
Corcorran (52)	1	1	1	0	1	4
Escudero et al. (25)	1	1	1	0	1	4
Galka et al. (32)	0	1	1	0	1	3
Jo et al. (35)	1	1	1	1	1	5
Kuo et al. (36)	1	1	1	1	1	5
Martin et al. (38)	1	1	1	1	1	5
McFarland et al. (39)	1	1	1	0	1	4
Metz et al. (40)	1	1	1	0	1	4
Peitzmeier et al. (41)	1	1	1	0	1	4
Roth et al. (43)	1	1	1	0	1	4
Roth et al. (44)	1	1	1	1	1	5
Roth et al. (45)	1	1	1	1	1	5
Schneider et al. (46)	1	1	1	0	1	4
Sherman et al. (47)	1	1	1	0	1	4
Stein et al. (48)	1	1	1	0	1	4
Tran et al. (49)	1	1	1	0	1	4
Walters et al. (50)	1	1	1	0	1	4
Walters et al. (51)	1	1	1	0	1	4

### PrEP continuum of care among WWID

#### PrEP awareness

Data on awareness of PrEP among WWID was reported in 14 studies (22, 24, 31, 32, 39–42, 44, 46, 50–53). Awareness of PrEP among WWID varied (range: 7–66%). Walters et al. (50) conducted a study among WWID in New York City, and showed that WWID who participated in transactional sex were more than three times more likely to be aware of PrEP than those who did not (aOR = 3.32; 95% CI = 1.22–9.0). In this same study, WWID who had a conversation about HIV prevention at syringe exchange programs were almost eight times more likely to be aware of PrEP than those who did not (aOR = 7.61; 95% CI = 2.65–21.84) (50). According to a study by McFarland et al. (39) among PWID in San Francisco (USA), WWID were more likely to be aware of PrEP than their male counterparts (63.4 vs. 52.7%, respectively, *p* = 0.025) (39). In a study on PrEP awareness among PWID in Philadelphia, injecting drug users that were aware of PrEP were more likely to be women (35.5 vs. 23.9%, *p* = 0.03) (44). In another study comparing PrEP awareness among various high-risk groups in New York, WWID had decreased odds of PrEP awareness compared to men who have sex with men (AOR: 0.18; 95% CI: 0.05–0.6) (51). In one study that examined awareness among individuals with opiate use disorder, there were no significant differences in PrEP awareness by gender (40).

#### PrEP knowledge

Knowledge of PrEP among WWID was assessed in three studies (31, 34, 39). In a study conducted by McFarland et al. (39), 38.9% of WWID knew that PrEP could prevent HIV transmission from sharing injection paraphernalia, and this knowledge did not differ between genders (39). Footer et al. examined PrEP knowledge among 16 WWID and female sex workers, and reported that knowledge was “low” among these populations, but this was not quantified (31). In contrast, in the study conducted among members of the International Network of People who use Drugs (INPUD) (34) most participants expressed that they had sufficient information on PrEP.

#### Access to PrEP care

Four studies examined access to PrEP care (33, 34, 43, 51). Notably, members of INPUD expressed the ethical concerns over providing WWID with knowledge about PrEP in settings where PrEP is not available (34). In Roth et al.'s study examining PrEP acceptance and access among PWID, 47.7% of WWID had seen a primary care physician in the past 6 months and 15.4% had been to an annual women's wellness exam (43). In the Walters et al. study among high-risk groups in New York City and Long Island, 25 and 32% of WWID, respectively, had exposure to HIV prevention professionals (50).

Regarding where WWID preferred to receive care, Roth et al. indicated that WWID preferred to be screened for HIV at the syringe exchange program rather than traditional sexually transmitted infection (STI) clinics. In particular, 90% of WWID indicated that they preferred HIV testing at a mobile van clinic (43). Similarly, members of INPUD also noted that community based services would be necessary for PrEP to be accessible to WWID given stigma toward PWID and the criminalization of drug use (33).

#### HIV risk perception

Four studies considered HIV risk perception (21, 24, 26, 54). Two studies quantitatively examined HIV risk perception among WWID (pooled sample size = 128) which averaged at 53.6% of individuals perceiving themselves to be at high risk of HIV (29, 48). In a PrEP demonstration study among WWID in Philadelphia, USA, participants indicated that periods of high drug consumption and engagement in transactional sex elevated their perceived risk of HIV. This also increased their desire to use PrEP (29).

In a qualitative study of WWID in Philadelphia, USA, women who were regularly engaged in harm reduction services had lower perceptions of HIV risk compared to women not engaged in such services. Overall, WWID were particularly concerned about obtaining HIV from sexual assault and accidental needlesticks, which positively impacted their decision to initiate PrEP (26).

In one survey examining HIV risk perception among people in prison in the United States, injecting drug use was positively correlated with perceived risk of HIV seroconversion in prison, and this relationship was slightly stronger among women than men (*p* < 0.01) (21). One study that examined awareness of and willingness to use PrEP among PWID and men who have sex with men (MSM) in India found that low perceived self-risk of HIV infection was the most common reason for being unwilling to use PrEP overall (24). Among those unwilling to use PrEP, 9% of WWID reported a lack of self-perceived HIV risk as the reason for their unwillingness.

#### PrEP acceptability

Data on PrEP acceptability among WWID were reported in 16 studies (22–26, 32–36, 40, 41, 43, 46, 48, 52). Results varied widely between studies (range: 23–100% acceptability). In the studies conducted among members of INPUD, PrEP was only acceptable if provided in a comprehensive package of harm reduction services as participants prioritized safe injection equipment over PrEP for HIV prevention.

In seven studies conducted among PWID in India (24) and the United States (35, 36, 40, 47, 48, 52), gender was not significantly associated with PrEP acceptability. However, in a study among PWID in Canada, WWID were more willing to use PrEP compared to men (OR 1.52, *p* = 0.028) (25). Similarly, in a study among individuals attending syringe exchange programs in New Jersey, WWID were more willing to use PrEP than their male counterparts (88.9 vs. 71.0%; *p* < 0.02) (43). Beyond gender, factors which influenced the acceptability of PrEP included concerns regarding side-effects (25, 33, 34, 43, 55), and access to health professionals (22, 34, 43). Participants who found PrEP more acceptable were those that engaged in sex work (25, 46) or transactional sex (43), had experienced sexual violence (41), had multiple recent sexual partners (25, 43), had other medical conditions (47), shared injection equipment (41, 46, 47), believed they were at high risk of HIV (48), and were of younger age (25, 41).

Results on the impact of injecting drug use on acceptability were mixed. In one study that examined PrEP acceptability among trans-women in Malaysia, injecting drug use was negatively associated with acceptability (*B* = −1.17, *p* = 0.001) (32). Interestingly, among female sex workers in Baltimore (USA), injecting drug use was not associated with acceptability of PrEP (41).

One study by Schneider et al. examined the acceptability of different forms of PrEP use and demonstrated higher acceptance of oral (62%) and arm-injection (60%) administration compared to implants (26%), vaginal gels (26%), vaginal rings (29%), abdomen injection (22%) and intravenous infusion (15%) among WWID (46).

#### PrEP usage

Data on the number of WWID that used or intended to use PrEP was collected in seven studies (22, 28, 38, 39, 46, 49, 54). In one study in Philadelphia intention to use PrEP was 88% among WWID. In this study, intention to use PrEP was associated with having fewer concerns discussing sexual history and drug use with their health provider (*p* < 0.01) (49).

Regarding usage of PrEP among PWID, there was no clear difference of PrEP use by gender across studies. Martin et al. found no significant difference in PrEP uptake by gender (OR 1.2, *p* = 0.16) in Thailand (38). However, McFarland et al. found that in San Francisco women were more likely to have used PrEP than heterosexual men (3.7% of women vs. 0% of heterosexual men, *p* = 0.007) (39).

Barriers to PrEP use among WWID included access to a doctor, homelessness, and potential theft of medication (22). Among WWID at a syringe service program in Philadelphia, factors that increased the odds of initiating PrEP included reporting sexual assault (*p* = 0.003), higher income (*p* = 0.06), frequency of syringe service programs attendance (*p* = 0.001), and inconsistent condom use (*p* = 0.03) (28).

#### PrEP adherence

Data on adherence to PrEP among WWID were collected in four studies. Across studies, adherence ranged from 5.6 to 52.3% (29, 37, 38, 45). In an analysis of 95 WWID in a PrEP demonstration project in Philadelphia, approximately half reported taking all PrEP medication at follow-ups, though prevention-effective levels were detected in only one participant urinalysis (45). Barriers to adherence included unstable housing and lack of storage for their medication. Adherence was also challenged by women's entrance to institutions that did not provide PrEP, such as some drug treatment and correctional facilities. Additionally, adherence depended on women's levels of drug-use and perceived HIV-risk at the time. When WWID felt at risk for HIV, they were more motivated to take PrEP. However, when WWID perceived they were at low risk of HIV (e.g., when abstaining from drug use) they discontinued use (29).

In the Bangkok Tenovir Study, which analyzed PrEP adherence in PWID by various demographic factors, women were more adherent compared to men (*p* = 0.006) (37). In the open-label extension of the Bangkok Tenovir Study, only 14% of WWID that returned for at least one follow-up visit had >90% adherence to PrEP. In the multivariable analysis, men were more likely to be adherent compared to WWID (OR = 1.9; 95% CI 1.0–3.6) (38).

#### Retention in PrEP care

Retention in PrEP care was assessed by the open-label extension of the Bangkok Tenovir Study (38) and the PrEP demonstration study in Philadelphia (45). The majority (69%) of Bangkok women returned for at least one follow-up clinic visit, but gender was not significantly associated with their likelihood of returning for a follow-up visit (38). In the PrEP demonstration study, retention fell in follow-ups at weeks 1 (93.7%), 12 (61.2%), and 24 (44.2%) among women in Philadelphia, and was most associated with access to syringe service programs (45).

#### PrEP communication

One relevant PrEP variable that was outside of the PrEP continuum of care but was mentioned in five studies was PrEP communication (27, 39, 40, 42, 54). In one study that considered the willingness to share information on PrEP among WWID, participants were willing to share information with 83% of people in their network (54). They were more likely to share information if the individual was homeless (UOR 3.3; 95% CI 1.5–7.6), an injecting drug user (UOR 2.3; 95% CI 1.1–4.7), engaged in transactional sex (UOR 4.5; 95% CI 1.6–12.5) or had a perceived high-risk of HIV (UOR 1.1; 95% CI 1.1–1.2). The study did not compare rates of sharing information between men and women. In another study examining PrEP communication among WWID, conversations having to do with PrEP occurred in 30/57 various relationships examined (27). In this study, individuals were motivated to have conversations of PrEP based on perceived HIV risk, gender similarity, to increase emotional connectedness and potential support from a peer, and when a negative outcome was perceived from not disclosing PrEP use.

Two studies considered PrEP communication in terms of conversations with the healthcare provider (39, 42). One study found no difference in gender in discussions of PrEP with healthcare provider (39). However, in another study examining drug treatment contexts and women's decision-making about PrEP a healthcare provider indicated that she never considered raising PrEP with heterosexual women clients (42).

## Discussion

This review of the PrEP continuum of care among WWID included 3,216 WWID across 32 studies. To our knowledge, this is the first systematic review stratified across the PrEP continuum of care to focus solely on this population, a highly vulnerable and marginalized population who are often overlooked in HIV research and prevention (5). WWID face several gender-specific challenges of drug use. Generally, WWID fall on the bottom of the hierarchy among PWID. This means that they may be forced to share needles or engage in risky income-generating behaviors to sustain drug use, such as sex work (56). This increases their risk for a variety of health harms, including higher mortality rates, levels of risky injecting, levels of risky sexual behavior, prevalence of blood-borne viruses, and psychological harm compared to men. WWID are also more likely to have a sexual partner who also injects drugs and be dependent on them for drugs compared to men (57). Many women who use drugs in such relationships also experience physical and psychological violence, which may preclude them from accessing harm reduction services, such as initiating PrEP uptake (57, 58). Furthermore, gender-based social responsibilities, such as child rearing, may prevent women from accessing health and harm reduction services generally. Notably, the fear of having children being apprehended may prevent WWID from accessing health services, including harm reduction services (59). As such, it is crucial to understand the gendered dynamics of injection drug use and harm reduction, an in particular, the PrEP continuum of care.

Despite the great need, there is no data from the ECDC on PrEP among WWID. In fact, data from ECDC show that more than 90% of current PrEP users in European countries belong to the MSM community (60). There is a strong need to scale up PrEP to other marginalized communities, such as WWID, if we are to reach the Sustainable Development Goals, and even in high-income countries with large-scale implementation (e.g., France, Netherlands, the United Kingdom, and the US), it is important to ensure that efforts are made to guarantee that these communities are reached at a sufficient scale. This review considered PrEP awareness (*n* = 14), PrEP knowledge (*n* = 3), access to PrEP care (*n* = 4), HIV risk perception (*n* = 4), PrEP acceptability (*n* = 16), PrEP usage (*n* = 7), PrEP adherence (*n* = 4), and retention in PrEP care (*n* = 1) among WWID. We also considered a new PrEP variable, PrEP communication (*n* = 5), that is highly relevant for improving awareness, knowledge, and usage in this population.

This review found that awareness, knowledge, and usage of PrEP in WWID is generally low. Suboptimal awareness was also found among other high-risk populations for HIV including women who use drugs at large (19, 61), women who engage in sex work (19), as well as MSM (62). However, WWID who were aware about PrEP were interested in its use, as PrEP acceptability was relatively high in most studies investigating it. Furthermore, acceptability was associated with HIV risk perception and engagement in high-risk sexual or injection practices. WWID are generally aware of and interested in lowering their risks of contracting HIV. However, as our review demonstrates, several structural issues challenge the ability of WWID to do so, including homelessness, sexual violence, unpredictability of drug use, and access to the healthcare system. A qualitative study by Felsher et al. (29) demonstrated that for some WWID, although there is a desire to use PrEP, it simply is overshadowed by other basic needs, such as access to food and shelter, generating an income and access to drugs. Whereas, one study performed in Kenya and South Africa showed drug use to be a PrEP-disrupting behavior (63), Felsher et al. showed that during periods where the women are not engaged in drug use, they are not as inclined to use PrEP as they feel their risk is lower (29). Risk of HIV transmission through non-injection routes (e.g., condomless sex) may also increase during periods of drug use (64).

The gap between PrEP acceptance and usage underscores the need for better provision of PrEP to WWID. WWID should be specified as a key population in PrEP technical guidelines, which is currently not the case in most countries, including in high-income countries (65). Further challenging PrEP awareness and usage in this population is the lack of engagement of WWID with the traditional healthcare system, as several studies in this review noted. This is unsurprising given the stigma, social inequality, and marginalization experienced by WWID, which leads to lack of healthcare access (66). As such, solutions to introduce PrEP at women's health clinics or other mainstream health services, as suggested by other research on women who use drugs (19), may fail to reach this population, as WWID indicated that they preferred to access care elsewhere.

Given these results, integrating PrEP services with low-threshold harm reduction and drug treatment services for PWID may be a more practical solution to engage this population in comparison to mainstream health services. In fact, members of INPUD highlighted that PrEP should only be administered as part of a comprehensive package of harm reduction (33, 34). Our findings align with previous research for engaging PWID in care (67–70). The studies in our review revealed that women that were more engaged in harm reduction services, such as syringe exchange programs, were more likely to be aware of and use PrEP. Furthermore, these services should have a holistic and gender-based approach to meet the unique needs and gender-based vulnerabilities of WWID, such as housing insecurity and sexual violence. In particular, integrating additional sexual health services in these settings could improve engagement in care for this population. In fact, a review of a pilot program which integrated reproductive healthcare within a needle and syringe program indicated that WWID were very satisfied with the services provided (71).

In addition to highlighting the need for integrating services, our results on PrEP communication underscore the role of peers in spreading knowledge and awareness about PrEP among WWID. Studies in this review indicated that WWID were very likely to share information on PrEP to other WWID, especially if they were deemed to be at high risk of HIV. This is in line with research on services for PWID which acknowledge the importance of engaging peers (68, 72, 73). For example, in Indonesia peer support was shown to help with HIV treatment initiation and adherence to HIV care among PWID. Furthermore, PWID were able to regain trust in the healthcare system and stay motivated to retain in HIV care (73). Similarly, in Senegal, researchers indicated that peer-led outreach among PWID could serve as an important part of harm reduction programs (72), Given their shared lived experiences, and potentially shared social networks, peers can help provide emotional and social support needed to engage with and maintain care. They can also diffuse harm reduction information through their social networks. However, rather than just communicating behavior change, peers can de-stigmatize drug use and encourage meaningful involvement of PWID in interventions aimed at improving their wellbeing (72). As such, harm reduction programs should involve peers provided Through a shared lived experience with adequate structural support to help generate trust and improve engagement in health and harm reduction services.

Our review highlighted several gaps in the evidence base. Most importantly, many studies (*n* = 21) were excluded because they did not stratify results by gender, which challenges understanding the needs of WWID, who may have different experiences compared to their male counterparts. It is crucial that future research on people who inject drugs disaggregate between men and women to improve service provision for both men and women. Alongside this, research and recruitment methods should be tailored to the needs of WWID to encourage their participation (e.g., female researchers in community-based settings). There was also a lack of geographical variation across studies, with most studies taking place in major metropolitan cities in the United States and an absence of studies from South America. Additionally, there was only one study that fitted our review criteria that examined transgender women. However, there is a great need for more research on trans WWID given that trans-women are at higher risk of contracting HIV (2). Lastly, very few studies examined PrEP adherence and retention among WWID, which are needed to improve engagement in care.

### Limitations

Several limitations exist in this systematic review. First, the number of studies and lack of geographic diversity limit the generalizability of our findings. This may have been as a result of our language restriction. Had we extended the inclusion criteria to other languages, the number of studies included in this review may have increased. The lack of geographic diversity meant we could not interpret differences in the PrEP continuum of care across different countries, or indeed, regions. Another intrinsic limitation of this review was the small proportion of studies on the PrEP continuum of care which included WWID and provided gender disaggregated data. In addition, when included, WWID were often only a small proportion of the study sample sizes. This also created difficulties in comparing results across studies. Therefore, this review and the findings within may change as more studies and reviews on the topic emerge over time.

## Conclusion

HIV research addressing the PrEP continuum of care under-recognizes the unique needs of and challenges faced by WWID, and especially transgender women. Steps of the care continuum, such as PrEP awareness and knowledge, may be improved by engaging WWID where they access health and/or social services, including in community and peer-based interventions. To improve PrEP usage and engagement in care among WWID, technical guidance should specify WWID as a key population for PrEP interventions. Furthermore, PrEP services could be integrated within gender-responsive harm reduction and drug treatment services as well as correctional services. Further studies are needed on PrEP retention and adherence among WWID, including in high-income countries where PrEP implementation has moved beyond demonstration projects to national programs.

## Data availability statement

The original contributions presented in the study are included in the article/supplementary material, further inquiries can be directed to the corresponding author/s.

## Author contributions

DG conceptualized this study. DG, TW, and JD scanned and assessed all potential articles for inclusion. DG drafted the manuscript with significant input from all other authors. All authors approved the final draft for submission.

## Funding

The project that gave rise to these results received support from a fellowship from La Caixa Foundation (ID 100010434) awarded to DG. The fellowship code is LCF/BQ/DI20/11780008.

## Conflict of interest

The authors declare that the research was conducted in the absence of any commercial or financial relationships that could be construed as a potential conflict of interest.

## Publisher's note

All claims expressed in this article are solely those of the authors and do not necessarily represent those of their affiliated organizations, or those of the publisher, the editors and the reviewers. Any product that may be evaluated in this article, or claim that may be made by its manufacturer, is not guaranteed or endorsed by the publisher.
